# Decrease in amygdala activity during repeated exposure to spider images predicts avoidance behavior in spider fearful individuals

**DOI:** 10.1038/s41398-020-00887-2

**Published:** 2020-08-20

**Authors:** Johannes Björkstrand, Thomas Agren, Andreas Frick, Olof Hjorth, Tomas Furmark, Mats Fredrikson, Fredrik Åhs

**Affiliations:** 1 Department of Psychology, Lund University, Allhelgona Kyrkogata 14M, 223 50 Lund, Sweden; 2 Department of Psychology, Uppsala University, Campus Blåsenhus, von Kraemers allé 1A, 752 37 Uppsala, Sweden; 3 Department of Neuroscience, Uppsala University, Akademiska sjukhuset, 751 85 Uppsala, Sweden; 4 Department of Clinical Neuroscience, Karolinska Institutet, Tomtebodavägen 18A, 171 77 Stockholm, Sweden; 5 Department of Psychology and Social Work, Mid Sweden University, Kunskapens väg 1, Östersund, Sweden

**Keywords:** Human behaviour, Learning and memory, Psychiatric disorders

## Abstract

Spider phobia is characterized by exaggerated fear of situations where spiders could be present, resulting in avoidance of such situations and compromised quality of life. An important component in psychological treatment of spider phobia is exposure to phobic situations that reduces avoidance behaviors. At the neural level, amygdala responses to phobic material are elevated, but normalizes following exposure treatment. To what extent amygdala activity decreases during a session of repeated phobic stimulation, and whether activity decrease is related to subsequent avoidance is not well studied. We hypothesized reduced amygdala activity during the course of repeated exposure to spider pictures, and that the degree of reduction would predict subsequent avoidance of spider pictures. To test our hypothesis, functional magnetic resonance imaging was performed in 45 individuals with spider fear during repeated exposure to spider pictures. Results showed that repeated exposure to spider stimuli attenuated amygdala reactivity and individual differences in activity reductions predicted subsequent avoidance behavior to spider pictures in an incentive-conflict task, with larger attenuations predicting less avoidance. At 6-month follow up, initial reductions in amygdala activation still predicted avoidance. This result demonstrates that reduction in amygdala responses is related to clinically meaningful outcomes in human anxiety, and suggests that within-session reductions in amygdala responses could be an important mechanism explaining the clinical effects of exposure therapy.

## Introduction

Specific phobia is a common disorder affecting around 15% of the population^1^. One of the most common subtypes of specific phobia is spider phobia with a prevalence of 3.5%^2^, which is characterized by severe fear reactions to situations involving spiders and avoidance of places and situations where spiders could be present^3^. Although effective treatments exist^4^, the neural mechanisms responsible for the treatment effect needs to be better understood.

At the neural level, fear and anxiety are regulated by the amygdala as has been demonstrated in animal studies^5^. Animal models of fear translate well to individuals with spider phobia who show elevated amygdala responses while viewing pictures of phobic stimuli as compared to individuals without the disorder^6–9^ The elevated amygdala response to phobic material can be reduced following cognitive behavior therapy (CBT), where graded exposure to spiders is a central component^8–11^. Not only does this treatment reduce amygdala activity but it also effectively reduces subjective ratings of spider fear and avoidance^4^. Previous brain imaging studies of neural effects of exposure-based treatment of spider phobia have generally measured brain responses before and after treatment but not during the exposure sessions^8–10^. Therefore, it is not known whether amygdala responses decrease during the actual exposure to spiders and if the hypothesized reductions in amygdala activity are directly related to clinically relevant outcomes, such as subsequent avoidance behavior of phobic material.

Within-session anxiety reductions during exposure sessions has previously been held to be crucial for the long-term behavioral effect of CBT for anxiety disorders and necessary for a favorable treatment outcome^12^. Lately this view has been challenged however, and it has been argued that habituation of fear responses within exposure treatment sessions is not important for overall treatment outcome and hence not predictive of long-term anxiety relief^13^. In these studies, within-session reductions in either physiological arousal (most commonly heart rate) or subjective ratings of anxiety or distress, are measured and then used to predict treatment outcome using an independent measure of clinical response (typically approach behavior or self-reported symptoms). In a review of the existing literature, Craske et al.^13^ noted that although physiological arousal and subjective fear ratings typically decrease during the course of an exposure session, these decreases do not consistently predict treatment outcome using independent measures, since only 3 out of the 7 reviewed studies found support for this claim, and concluded that within-session fear habituation is not an important component of exposure therapy. However, as noted, this stance is based on evidence from studies quantifying fear habituation during exposure sessions using psychophysiological measures or self-reports of subjective distress or anxiety. It is not known how within-session changes in neural activations relate to clinically meaningful behaviors such as avoidance. To evaluate whether changes in neural responses predict clinically meaningful outcomes, it would be of importance to investigate if alterations in amygdala responses during repeated exposure to phobic material can predict subsequent avoidance behavior over short and long time periods. Of note, a small (n = 11) positron emission tomography (PET) study^14^ has found that repeated exposure to phobia relevant cues (spider pictures) lead to within-sessions reductions in amygdala activity above and beyond what could be observed for neutral cues. Here we investigate whether this finding can be replicated in a larger sample using functional magnetic resonance imaging (fMRI). Critically, we also investigate whether the hypothesized amygdala reductions can predict later approach behavior to phobia relevant cues, a clinically meaningful outcome measure, over the short and long term.

**Fig. 1:**
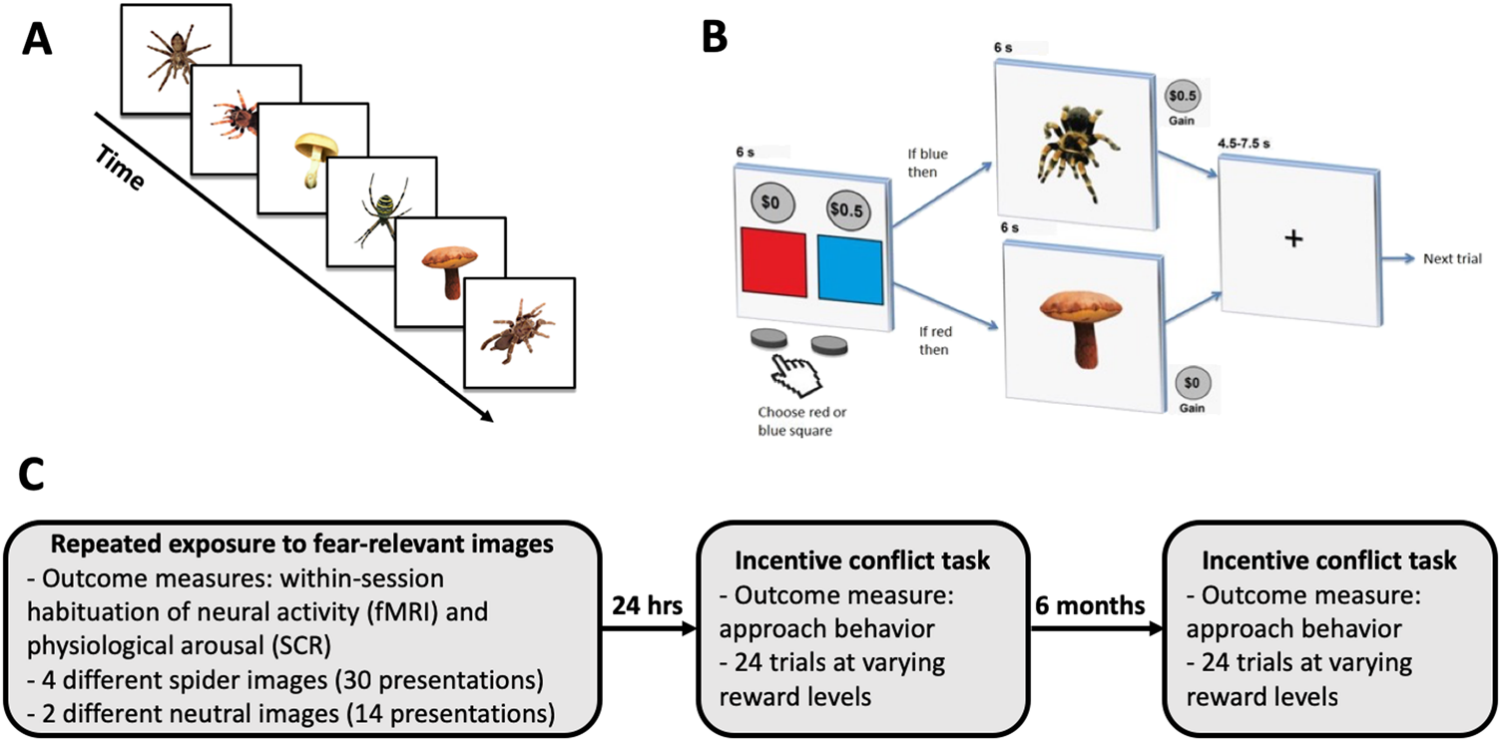
Experimental design. **a** Spider fearful subjects (*n* = 45) underwent brief repeated exposure to fear-relevant images (spiders) interleaved with neutral control images (mushrooms), while we measured neural activity and physiological arousal. **b** After 24 h (*n* = 45) and at 6-month follow-up (*n* = 40), subjects completed an incentive conflict task measuring approach behavior of fear-relevant cues. During this task subjects repeatedly chose whether to watch a spider picture and receive a small monetary reward, or watch a neutral image and forego reward. **c** During repeated exposure we quantified within-session habituation for neural activity and physiological arousal by contrasting responses during early trials against late trials. These measures where then used to predict approach behavior at 24 h and 6 months.

In the present study, we first evaluated if repeated exposure to fear-inducing spider pictures in individuals with phobic level fear of spiders resulted in reduced amygdala reactivity and physiological arousal within a single brief session of repeated exposure to phobia-relevant cues, see Fig. 1. To accomplish this, participants were exposed to repeated presentations of spider pictures while in an MRI scanner, and as a control for generally diminished reactivity to visual stimulation we included neutral slides of mushrooms. Simultaneously, we also measured event-related skin conductance responses (SCRs) to quantify within-session changes of physiological arousal. Subsequently, after 24 hrs and 6 months, participants completed a behavioral approach-avoidance conflict task where they could choose to accept monetary incentives to view a spider picture or to avoid it without earnings. We predicted stronger exposure-induced reductions of amygdala activity and physiological arousal to spider than to neutral cues. Additionally, we predicted that reductions in amygdala reactivity to spiders would be associated with more approach behavior in the subsequent incentive conflict task, whereas reductions in physiological arousal would not. We further performed a whole-brain analysis to explore if other brain regions showed reduced responses as a function of repeated phobic stimulation, and similarly investigated whether other brain regions could also predict subsequent approach behavior.

## Methods

### Participants

One hundred and thirty individuals responded to public advertisements searching for spider fearful subjects and completed a web-based version of the validated Spider Phobia Questionnaire (SPQ)^15,16^. Based on their self-reported fear of spiders, 49 high-scoring individuals were enrolled in the study. To ensure that subjects could undergo magnetic resonance imaging safely, exclusion criteria were: prior surgery to heart or the brain, implanted medical devices (e.g. pacemakers or a medicine pump), metal implanted in body, self-reported claustrophobia, and pregnancy. Also, those currently using psychotropic medication where excluded. Of the 49 enrolled, 45 subjects were included in the final analysis. One subject discontinued the experiment upon entering the scanner because of a claustrophobic reaction, one discontinued during scanning due to a panic attack elicited by spider picture presentation, and two subjects were excluded from analysis due to incomplete or faulty behavioral data. Of these, one subject misunderstood the instructions of the behavioral task and one subject discontinued the behavioral task due to muscle cramps. This subject did however complete the behavioral task at the follow-up measurement and is included in these analyses. Thus, 45 individuals (34 women) completed the initial study protocol. At the 6-month follow up 40 of the initial 45 subjects (89%) were able to participate. All subjects scored above the 95^th^ percentile on the SPQ, being 19.36 ± 2.98 (mean ± S.D.). Age was 26.16 ± 7.58 years and all had been markedly afraid of spiders for a minimum of 10 years with average duration being 20.21 ± 8.04 years. The study was approved by the Regional Ethical Vetting Board in Uppsala and written informed consent was obtained from all subjects, Subjects received 1000 Swedish Crowns (SEK) for participation (1 SEK roughly equal to 0.11 dollar) in addition to their earnings obtained during the behavioral task. Data has been partly presented in two previous studies^17,18^ investigating reconsolidation effects in this cohort, whereas here we perform new analyses to evaluate within-session effects of repeated spider presentations on neural activation and physiological arousal, as well as it’s relationship to approach avoidance behavior 24 h and 6 months later.

### Stimuli

For the repeated-exposure protocol, spider pictures, all depicting a spider on a white background, were chosen from a previous study^19^. To ensure that the experimental effects were not dependent on specific stimuli, we created eight stimulus sets, each containing differing combinations of four different spider pictures, and each subject was then randomly assigned one of these stimulus sets for the exposure. To evaluate general response diminution as a function of visual stimulus repetition, but unrelated to fear, two neutral pictures depicting mushrooms, also obtained from the same study^19^, were interleaved with spider pictures. For the behavioral approach-avoidance test we used five spider and five mushroom pictures, but no picture used in the behavioral test was shown during the exposure. Timing of cue presentation during repeated exposure and cue selection during the behavioral test was controlled using E-prime 2.0 (Psychology Software Tools, Pittsburgh, PA).

### Procedure

First, participants signed the informed consent form and were then placed in the MR-scanner in a supine position and underwent the repeated exposure to phobia relevant images while we collected functional brain imaging data.

#### Repeated exposure to spider pictures

Images were presented through video-goggles mounted on the head-coil. During repeated exposure, four different spider pictures were presented 30 times in total, in randomized order, with two neutral slides depicting mushrooms presented 14 times in total, interleaved with spider pictures. Each picture was shown for 6 s with a mean average inter trial interval of 6 s, varying between 4.5 and 7.5 s. After the exposure, anatomical reference images were collected, and subjects dismissed.

#### Incentive conflict task

Participants returned 24 h following the fMRI scanning to perform the incentive conflict task, which was also repeated ~6 months after the initial experiment. During this behavioral approach-avoidance test, participants could choose to view a fear-relevant spider picture and receive a varying monetary reward or view a neutral picture without payment (see Fig. 1b. for a schematic design). Participants were shown a slide with a red and a blue square appearing side-by-side on a white background. Subjects were informed that if they chose the blue square, by pressing the associated response key, a spider picture would appear on the screen after a short delay, whereas if they chose the red square, a picture of a mushroom would follow (colors were counterbalanced across subjects). Subjects were also informed that the task would include new spider pictures, not seen before. Responses were given using handheld response buttons. In order to improve the accuracy of the behavioral metric, varying degrees of monetary reward were included to motivate approach to spider cues as we have done in a previous approach-avoidance task^20^. Above each square a gray circle appeared that displayed the amount of money received given that a particular square was chosen. The amount associated with choosing the cue leading to the presentation of a neutral picture was always 0 SEK and the amount for choosing the cue leading to a fear relevant picture varied between, 0, 0.1, 0.5, 1, 2, and 5 SEK. Subjects completed four trials at each reward level adding up to a total of 24 trials presented in randomized order. The slide during which subjects made their choice was shown for 6 s and the response could be made any time during this interval. Then, contingent on the response either a mushroom or a spider picture appeared on the screen for 6 s. Between each trial there was a fixation cross with an average duration of 6 s, varying between 4.5 and 7.5 s.

### Physiological arousal

To quantify within-session changes in physiological arousal, even-related SCRs were measured using two Ag–AgCl electrodes (EL509 Biopac electrodes) filled with isotonic electrode gel (GEL101 Biopac gel) attached to the palmar surface of the hypothenar eminence of the left hand. SCRs were amplified and recorded with a sampling rate of 1000 Hz using the Biopac MP150 system and Acqknowledge software, version 4.2 (BIOPAC Systems, Goleta, CA, USA). Subsequently, a low-pass filter of 0.5 Hz was applied to the skin conductance signal in order to filter out high frequency artefacts. Then, similar to previous studies^21–23^, event-related SCRs were extracted using a local baseline approach, by deducting the mean value of the skin conductance signal 1.2–1.5 s after stimulus onset from the peak value 1.5–4.5 s after stimulus onset. To reduce between subject variability, SCRs were then range-corrected by dividing each response by the largest stimulus elicited SCR for each individual subject^24^. For comparability, within-session changes in SCRs was analyzed in the same way as brain responses (see below).

### Brain imaging

Data were acquired using a 3T whole body MR scanner (Philips Achieva 3.0T TX, Philips Medical Systems, Best, The Netherlands) with a 32-channel head coil. Head movement was restricted using foam cushions. Initial scanning was performed to create an anatomical T_1_-weighted reference data set to a voxel size of 0.5 × 0.5 × 1.0 mm and 170 slices. During visual presentations, blood-oxygen-level-dependent (BOLD) imaging was performed using a single shot EPI sequence with parameters TE/TR 35/3000 ms, flip angle 90°, acquisition matrix 76 × 77, acquired voxel size 3.0 × 3.0 × 3.0. A total of 33 slices with a 1-mm gap were sampled for whole-brain coverage. Pre-processing of functional imaging data included slice time correction, motion correction, co-registration to anatomical images, normalization to MNI-space, and smoothing with an 8-mm FWHM kernel.

### Regions of interest and statistical analysis

Data were evaluated using SPM 8 (Welcome Department of Cognitive Neurology, University Collage, London, http://www.fil.ion.ucl.ac.uk) and a region of interest (ROI) approach with amygdala definitions taken from the Talairach and Tournoux atlas^25–27^. Results were considered significant if they survived correction for multiple comparisons using small-volume correction (SVC) by applying family wise error correction (FWE) within the specified ROI. We also performed exploratory whole-brain analysis, where results were considered significant if they survived correction for multiple comparisons at an alpha level of *p* < 0.05 after whole-brain FWE-correction. Only activations that exceeds these thresholds are reported and displayed in figures. Brain images in the figures were created using MRIcroGL (available at https://www.nitrc.org/projects/mricrogl/). For illustrative purposes, clusters are smoothed using the built-in smoothing tool in MRIcroGL.

To evaluate changes in brain activity and physiological arousal during the course of repeated exposure, we compared activity elicited by the first eight spider picture presentations to the last eight presentations. To determine whether effects were specific to phobia relevant pictures we performed corresponding analyses for neutral pictures, comparing the first four with the four last picture presentations. To evaluate the stimulus by time interaction on voxel-wise neural activation we created a contrast by subtracting the early>late contrast for neutral pictures from the early>late contrast for spider pictures. To corroborate this analysis, we also created contrasts of the first and last eight picture presentation for spiders over baseline activity (fixation cross) as well as for the first and last four neutral picture presentations and then extracted the average beta-values from the left and right amygdala ROIs. We then entered these values into a 2 × 2 repeated measures ANOVA with factors stimuli (fear-relevant vs neutral) and time (early; late), and a corresponding analysis was performed for SCRs.

With regard to the behavioral measure, a mean approach score was calculated for each subject reflecting the proportion of trials the subject chose to view a fear relevant cue instead of a neutral cue, averaged over all trials and reward levels. For example, an approach score of 0.5 indicates that the subject chose to view a spider on 50% of all trials. To perform brain-behavior correlations, approach scores were entered as a regressor into the SPM-model used to evaluate brain activity reductions (the early>late contrast for spider pictures), as described above, and then we performed a voxel-wise regression analysis. To investigate if the effect is specific to phobia relevant cues, the corresponding analysis was also performed for neutral cues (the early>late contrast for mushroom pictures). Thus, the correlation analyses reflect the strength of the linear association between reductions in neural activation during exposure and subsequent approach behavior. Analyses investigating the association between reductions in physiological arousal and subsequent approach behavior, was performed in a similar way, where we created a habituation index by subtracting SCRs during the last eight spider image presentations from SCRs during the first eight spider image presentations, and then used this metric to perform regression analyses with approach behavior. Apart from voxel-wise analysis of neural activation, JASP (version 0.9.2) was used for all statistical analysis.

## Results

### Within-session changes in brain activity

**Fig. 2:**
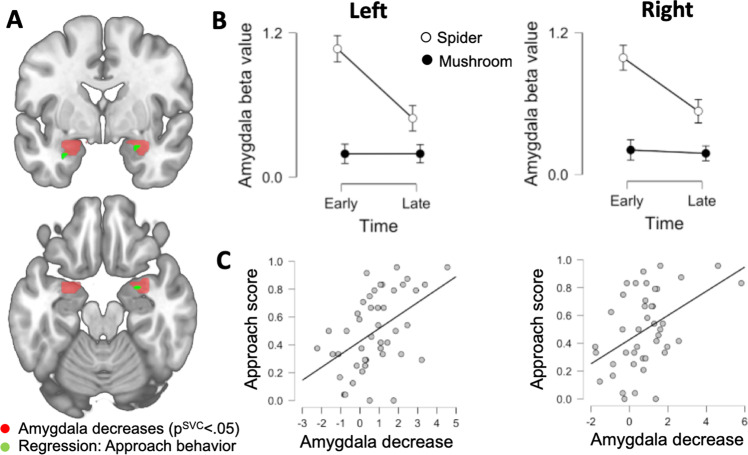
Amygdala activity decreases during repeated exposure and predicts approach behavior. **a** Red colors show voxels in the amygdala where we found significant reductions in neural activity when comparing early to late trials (stimulus by time contrast). Green colors show voxels in the amygdala where we found significant association between amygdala reductions to fear relevant cues (spider: early>late contrast) and approach behavior during the incentive conflict task 24 h after repeated exposure. Results are displayed on the standard MNI template. **b** Using extracted average beta-values for the entire amygdala ROIs we found significant decreases for fear-relevant but not neutral cues in both the left and right amygdala. Points and error-bars show mean and SEM. **c** Scatterplots show the correlation between left and right amygdala decreases respectively and approach behavior at 24-h time point. The amygdala values reflect decreases (spiders: early>late contrast) in the peak voxel from the voxel-wise regression analysis. The association was significant and similar in the left and right amygdala (left: *r* = 0.48; *p* < 0.001; right: *r* = 0.45; *p* = 0.002).

Significantly higher amygdala activation was observed during early as compared to late spider trials during repeated exposure (Left: *xyz*; −30, −1, −20; *Z* = 4.08*; p*^SVC^ = 0.001; 1269 mm^3^; Right: *xyz*; 30, 2, −20; *Z* = 3.99; *p*^SVC^ = 0.001; 1107 mm^3^). The corresponding analysis for neutral stimuli did not reveal any significant voxels. To evaluate the stimulus by time interaction we created a contrast by subtracting the early>late contrast for neutral pictures from the early>late contrast for spider pictures, see Fig. 2a. We found large reductions in the amygdala bilaterally (Left: *xyz;* −30, 2, −20; *Z* = 4.06; *p*^SVC^ = 0.001; 1647 mm^3^; Right: *xyz*; 27, 2, −20; *Z* = 4.71; *p*^SVC^ < 0.001; 1566 mm^3^) indicating that amygdala reactivity decreases specifically as a function of exposure to fear-inducing phobia-related stimuli and not merely reflecting effects of repeated visual stimulation. To complement this analysis, we extracted the average beta-values from the entire left and right amygdala ROIs and entered these values into a 2 × 2 × 2 repeated measure ANOVA with stimuli (fear-relevant; neutral), time (early; late), and side (left; right) as factors, see Fig. 2b. Similar to the voxel-wise analysis we found a significant stimuli by time interaction (*F*(1, 44) = 11.61; *p* = 0.001) as well as significant main effects of stimuli (*F*(1,44) = 29.45; *p* < 0.001) and time (*F*(1,44) = 8.24; *p* = 008). No difference in activation was observed between the right and left amygdala as indicated by a non-significant main effect of side (*F*(1,44) = 0.05; *p* > 0.800), nor was any interaction with side significant (all *p’s* > 0.1) indicating similar effects bilaterally in the amygdala. Simple main effects analysis, with time as the simple effect factor and stimuli and side as moderator factors, comparing early and late trials for fear-relevant and neutral cues respectively revealed a significant decrease in amygdala activity for spider pictures (left: *F*(1,44) = 7.74; *p* = 0.008; right: *F*(1,44) = 16.13; *p* < 0.001) but not neutral pictures (left: *F*(1,44) = 0.10; *p* *=* 0.760; right: *F*(1,44) = 0.0001; *p* = 0.990).

**Fig. 3:**
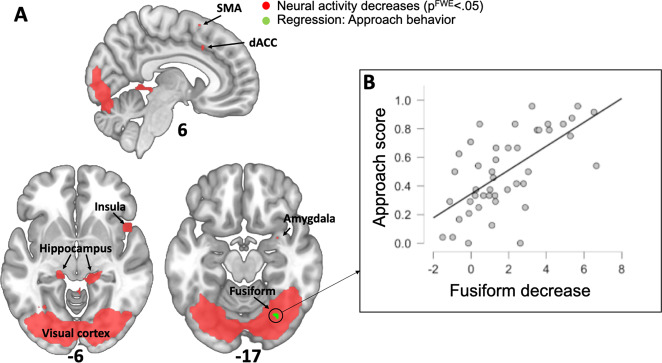
Whole-brain analysis of within-session decreases in neural activity and association with approach behavior. **a** Red colors show voxels where we found significant reductions in neural activity when comparing early to late trials (stimulus by time contrast). Green colors show voxels where we found a significant association between neural activity reductions to fear relevant cues (spider: early>late contrast) and approach behavior during the incentive conflict task 24 h after repeated exposure. Results are displayed on the standard MNI template. **b** Whole-brain regression analysis revealed a cluster in the right fusiform gyrus that predicted approach behavior (depicted in green). The scatter plot shows the association between extracted beta-values from the peak voxel in this cluster (spider: early>late contrast) and approach behavior at the 24-h time point. The association was strong and significant (*r* = 0.64; *p* < 0.001).

Whole-brain analyses revealed reduced spider related activation (the stimulus by time interaction contrast) passing correction for multiple comparisons in large portions of the occipital lobe encompassing the fusiform gyrus. Reduced activation was also observed in the anterior insula, posterior hippocampus, amygdala, and the supplementary motor area (see Table 1 and Fig. 3a). No increase in neural activity passing the statistical threshold was observed for this contrast when late trials were compared to early trials.

**Table 1:** Whole-brain analysis showing greater habituation of brain responses to spiders than mushrooms (time by stimulus contrast) at *p* < 0.05 family-wise-error corrected.

Region (AAL label)	Coordinates (*x*, *y*, *z*)	*z*-value	Volume (mm^3^)
Left occipital mid	−21, −94, 4	8.04	27,756
Right hippocampus	18, −31, −5	5.58	585
Left hippocampus	−15, −31, −5	5.17	225
Right insula (anterior)	48, 14, −5	4.96	234
Right amygdala	27, 2, −20	4.71	27
Right calcarine sulcus	30, −64, 16	4.62	18
Mid cingulum	6, 17, 37	4.62	36
Supplemental motor area	6, 14, 61	4.60	9

Coordinates are given according to the MNI-coordinate system, *z*-value reflects peak voxel activation and volume indicates size of cluster.

### Within-session changes in physiological arousal

**Fig. 4:**
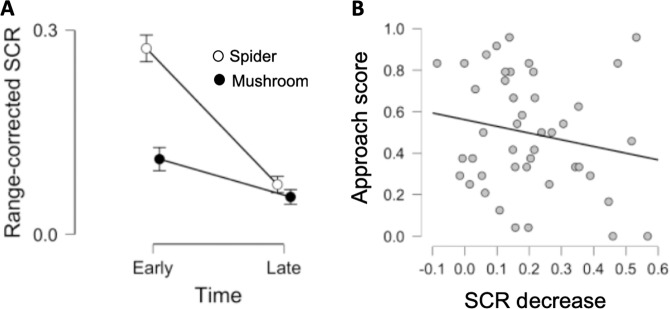
Event-related physiological arousal decreases during repeated exposure but does not predict approach behavior. **a** Physiological arousal, measured with event-related SCRs, decreased for both fear-relevant and neutral cues, but decreases were greater for fear relevant cues, indicated by a highly significant stimulus by time interaction. Subjects showed higher SCRs to fear relevant cues compared to neutral cues during early but not late trials of repeated exposure. Points and error-bars denote means and SEMs. **b** There was no significant relationship between decreases in physiological arousal to fear-relevant cues and approach behavior. The scatter plot shows the association between decreases in SCR (spiders: early>late) and approach behavior at the 24-h time point.

To investigate the effect of repeated exposure to phobia relevant images on physiological arousal we extracted SCRs to fear relevant and neutral images during early and late trials and entered these values into a 2 × 2 repeated measure ANOVA with stimuli (fear-relevant; neutral) and time (early; late) as factors, see Fig. 4a. Similar to analysis of amygdala responses we found a significant stimuli by time interaction (*F*(1,44) = 25.72; *p* < 0.001) as well as significant main effects of stimuli (*F*(1,44) = 35.69; *p* < 0.001) and time (*F*(1,44) = 61.11; *p* < 0.001). Simple main effects analysis, with time as the simple effect factor and stimuli as the moderator factor, comparing early and late trials for fear-relevant and neutral cues separately revealed a significant decrease in SCRs for spider pictures (*F*(1,44) = 71.49; *p* < 0.001) as well as neutral pictures (*F*(1,44) = 8.12; *p* = 0.007), with decreases for spider pictures being significantly greater than for neutral pictures as indicated by the significant stimulus by time interaction. Simple main effects analysis, with stimulus as the simple effect factor and time as the moderator factor, showed significantly larger SCRs for spiders as compared to neutral cues during early (*F*(1,44) = 35.51; *p* < 0.001) but not late trials (*F*(1,44) = 2.77; *p* = 0.100). Similar to the analyses on neural responses, overall, the results confirm large decreases in physiological arousal to fear relevant cues above what could be observed for neutral cues.

### Approach-avoidance behavior

During the first behavioral test the entire sample had a mean approach proportion of 0.497 ± 0.041 (mean ± SEM). At the 6-month follow-up, the whole sample had a mean approach proportion of 0.418 ± 0.041. Behavior during the first and second avoidance test was significantly correlated (*r* = 0.682; *p* < 0.001; *n* = *40*), demonstrating adequate test–retest reliability over 6 months. To evaluate whether approach proportion changed over time, we performed a paired *t*-test comparing the 24-h and 6-month time points. The results show a small but significant decrease in approach proportion over time (*t* = 2.03; df = 39; *p* = 0.049; *d* = 0.32), indicating a slight relapse in avoidance behavior over 6 months.

### Regression analyses: relations between within-session habituation and approach behavior

Voxel-wise regression analysis restricted to the amygdala ROIs showed that reductions in responses to spider pictures from early to late trials predicted approach behavior 24 h later during the incentive conflict task (left: *xyz*; −30, −4, −23; *Z* = 3.29; *p*^SVC^ = 0.013; 108 mm^3^; right: *xyz*; 24, −4, −23; *Z* = 3.05; *p*^SVC^ = 0.029; 81 mm^3^), with larger amygdala reductions during repeated exposure being associated with more approach behavior. Clusters both in the left and right amygdala were co-located with voxels where we observed significant decreases, see Fig. 2a, c. We performed the corresponding analyses for the neutral pictures but did not find any voxels with a significant relationship between decreased amygdala responses and approach behavior, indicating that this association is specific to fear-related stimuli.

Whole-brain analyses revealed a small cluster located in the right fusiform gyrus (*xyz*; 27, −67, −17; *Z* = 4.73; *p*^FWE^ = 0.031; 81 mm^3^) where we observed a significant correlation between avoidance and change in responses to spider pictures from early to late trials surviving correction for multiple comparisons. This cluster was co-located with regions where we found significant decreases in neural activations (see Fig. 3). The corresponding analysis for the neutral pictures did not reveal any significant voxels.

To evaluate whether reduction of amygdala activity within a session predicted approach behavior over a longer time period, we also related reductions in amygdala activity to approach behavior during the incentive conflict task after 6 months. We found a significant relationship between left, but not right, amygdala decreases and approach behavior (left: *xyz*; −27, −1, −26; *Z* = 3.03; *p*^SVC^ = 0.028; 27 mm^3^), which were co-located with voxels where we found an association for the 24 h time-point. To investigate specifically whether voxels that predicted approach behavior after 24 h, still did so after 6 months we repeated the analysis, but restricting the ROI only to voxels where there was a significant association at the 24 h time-point. In the left amygdala we found that all voxels in the cluster still showed a significant association (left: *xyz*; −27, −1, −26; *Z* = 3.03; *p*^SVC^ = 0.003; 108 mm^3^), whereas in the right amygdala 2 out of 3 voxels showed a significant association (right: *xyz*; 27, −1, −20; *Z* = 2.16; *p*^SVC^ = 0.027; 54 mm^3^). Whole-brain analysis did not reveal any significant correlations between approach behavior at the 6-month follow-up and change in neural responses to spider pictures from early to late trials surviving correction for multiple comparisons (whole-brain correction; *p*^FWE^ < 0.05). When restricting the analysis to the voxels where we found a significant correlation for the 24 h time-point and applying small-volume correction we found a significant association encompassing the entire cluster (*xyz*; 30, −70, −17; *Z* = 2.36; *p*^SVC^ = 0.017; 81 mm^3^) although with a weaker effect.

To evaluate whether within-session reductions in physiological arousal predicted subsequent approach behavior, the SCR-habituation index (reflecting changes in SCRs from early to late trials for spider pictures) was correlated against approach behavior at 24 h and 6 months respectively. The results showed that reductions in physiological arousal was not related to approach behavior either at 24 h (*r* = −0.19; *n* = 45; *p* = 0.220) or 6 months (*r* = −0.14; *n* = 40; *p* = 0.378), see Fig. 4b.

## Discussion

In this study, we tested, and found support for, the hypothesis that repeated exposure to phobia-relevant cues leads to within-session attenuation of amygdala activity in spider fearful subjects, in line with a previous PET study^14^. This finding is also consistent with previous studies^8–11^ showing reductions in amygdala activity to phobic images following exposure therapy for spider phobia, indicating that within-session reductions mirrors between-session reductions as far as amygdala activation is concerned. This hints at the possibility that within-session habituation of amygdala activity could be a predictor of, and perhaps even a prerequisite for between-session reductions, although we are unable to directly address this question in the present study.

Moreover, larger reductions in amygdala activity predicted less avoidance behavior in an incentive conflict task both 24 h later and at 6-month follow-up, whereas reduction of physiological arousal did not. These findings show that within-session reductions in amygdala responses to phobic stimuli is related to avoidance behavior over long periods, consistent with a growing body of literature linking bottom-up processes from the amygdala to therapeutic success^9–11,28–31^. Similarly, a previous neuroimaging treatment study of exposure therapy has shown that pre-post reductions in amygdala activity are correlated to pre-post decreases on a self-report questionnaire evaluating clinical outcome^9^. Our results indicate that similar to between session reductions^9^, this association also holds for within-session reductions in amygdala responses. Previous studies have indicated that within-session fear habituation (indexed by physiological arousal or subjective ratings) are not a crucial component of exposure-based interventions^13^. Our findings are largely in line with these observations, but further suggests that although within-session reductions in physiological arousal are not predictive of later approach behavior, habituation of amygdala responses are, and thus could be an important treatment mechanism in exposure therapy, such that successful treatment outcome may be dependent on adequate within-session reduction of amygdala responses.

In an exploratory whole-brain analysis we also found large reductions in neural activity as an effect of repeated exposure to phobic images in the occipital cortex, dorsal hippocampus, anterior insula, dorsal ACC, and supplementary motor area. A previous meta-analysis of treatment studies in spider phobia^8^ has indicated that reductions in activity following treatment are consistently noted in the amygdala, anterior insula, supplementary motor area, dACC, thalamus, and dlPFC. Similarly, a later study showed reductions in the amygdala, anterior insula, and dACC immediately following exposure therapy for spider phobia, and further reductions in visual cortex activation at a 6-month follow-up^10^. These areas correspond well with areas that are activated when phobic stimuli are compared to neutral stimuli^8,9^. Therefore, it seems that brain areas that show elevated activation to spiders in individuals with phobia normalize their responses following treatment. The pattern of within-session reductions in spider related activity that we observed are largely consistent with previous studies comparing brain responses to spiders before and after treatment^8,10^, indicating that in the context of neural activity, within-session reductions correspond well to between-session reductions. One clear difference, however, was that the strongest reduction in activity in our study was found in the occipital lobe covering large areas of relevance to vision, a small portion of which correlated with avoidance behavior at the 24 h post-test, although no exstensive reductions in visual areas were reported in the meta-analysis by Ipser et al.^8^ and Lipka et al.^9^ reported only trend-level reductions in the fusiform gyrus as an effect of treatment. Hence, it seems that although the over all pattern of reductions in neural responses correspond well from within-session to between-session, there might be regional differences in the occipital cortex. Notably, although Hauner et al.^10^ did not find any reductions in visual cortex activation immediately after treatment they observed large reductions at a 6-month follow-up, such that exposure therapy had little immediate effect on visula cortex activation which then appeared after a delay. This could account for the lack of decreases in occipital cortex activation in the previous meta-analysis^8^ which did not include long-term follow-up data. This is interesting since it suggests that within-session habituation in neural activity better reflect long-term rather than short-term between-session changes in neural activation.

Apart from the amygdala, we further found that reactivity reductions in the right fusiform cortex to spider pictures was correlated with approach behavior, meaning that larger reductions in activation of the fusiform cortex predicted less avoidance. The finding is in accordance with Hauner et al.^10^ who reported that post-treatment activity to spider stimuli in the right ventromedial occipital cortex predicted reductions in self-reported fear following exposure therapy. This suggests that within- and between-session reductions in neural activation of the ventral visual stream may be of importance to emotional experience and behavioral reactions to fearful stimuli.

Although the results were in line with our hypotheses and consistent with previous findings there are some limitations that need to be taken in to account when interpreting the results, specifically in relation to the generalizability of the present findings. This was an experimental study performed on a clinical analog sample. Subjects were recruited from a community sample and selected for inclusion based on high-scores on a validated spider fear questionnaire, but were not formally diagnosed with specific phobia using structured interviews. The repeated exposure protocol was brief and included only still-frames of spiders such that the intervention is likely insufficient to produce clinically meaningful changes in spider phobia. The study was designed this way to achieve a high degree of experimental control, and to facilitate data-collection, but whether these effects can be generalized to contexts more closely resembling clinical treatment of specific phobia and other anxiety disorders remain an area for further study.

In summary, brief repeated exposure to phobia relevant images in spider fearful subjects, resulted in reduced amygdala activity and physiological arousal above and beyond what could be observed for neutral images. Larger amygdala attenuations predicted less avoidance of feared cues over both 24 h and 6 months, whereas attenuation of physiological arousal did not. Thus, the current results indicate that amygdala alterations predict approach behavior over long time spans suggesting that within-session reductions in amygdala responses may be important for long-term behavioral change, which is the ultimate goal of behavior therapy.
